# Changes in Expression Pattern of Selected Endometrial Proteins following Mesenchymal Stem Cells Infusion in Mares with Endometrosis

**DOI:** 10.1371/journal.pone.0097889

**Published:** 2014-06-05

**Authors:** Lisley I. Mambelli, Rodrigo C. Mattos, Gustavo H. Z. Winter, Dener S. Madeiro, Bruna P. Morais, Eduardo Malschitzky, Maria Angélica Miglino, Alexandre Kerkis, Irina Kerkis

**Affiliations:** 1 Laboratório de Genética, Instituto Butantan, São Paulo, SP, Brasil; 2 Programa de Pós-Graduação em Anatomia dos Animais Domésticos e Silvestres da Faculdade de Medicina Veterinária e Zootecnia da Universidade de São Paulo, São Paulo, SP, Brasil; 3 Reprolab, Faculdade de Medicina Veterinária, Universidade Federal do Rio Grande do Sul, Porto Alegre, RS, Brasil; 4 Curso de Medicina Veterinária, ULBRA, Canoas, RS, Brasil; University of Lyon, France

## Abstract

Mesenchymal stem cells (MSCs) due to their self-renewal potential and differentiation capacity are useful for tissue regeneration. Immunomodulatory and trophic properties of MSCs were demonstrated suggesting their use as medicinal signaling cells able to positively change local environment in injured tissue. Equine endometrosis is a progressive degenerative disease responsible for glandular alterations and endometrial fibrosis which causes infertility in mares. More precisely, this disease is characterized by phenotypic changes in the expression pattern of selected endometrial proteins. Currently, no effective treatment is available for endometrosis. Herein, we aimed at the evaluation of expression pattern of these proteins after allogeneic equine adipose tissue-derived multipotent mesenchymal stem cells (eAT-MSCs) infusion as well as at testing the capacity of these cells to promote endometrial tissue remodeling in mares with endometrosis. eAT-MSC (2×10^7^/animal) were transplanted into mares’ uterus and control animals received only placebo. Uterine biopsies were collected before (day 0) and after (days 7, 21 and 60) cells transplantation. Conventional histopathology as well as expression analysis of such proteins as laminin, vimentin, Ki-67-antigen, α-smooth muscle actin (α-SMA) and cytokeratin 18 (CK18) have been performed before and after eAT-MSCs transplantation. We demonstrated that eAT-MSCs induced early (at day 7) remodeling of endometrial tissue microenvironment through changes observed in intra cellular and intra glandular localization of aforementioned proteins. We demonstrated that eAT-MSCs were able to positively modulate the expression pattern of studied secretory proteins as well as, to promote the induction of glandular epithelial cells proliferation suggesting local benefits to committed endometrial tissue environment after eAT-MSCs transplantation.

## Introduction

Equine endometrosis is a degenerative disease of uterine glands and surrounding stroma which leads to infertility [1], [2]. In mares, the trophoblast is non-invasive and uterine glands secretions are considered essential to embryo implantation, fetal development and survival. According to [3], endometrosis is defined as an active (can produce all secreted proteins) or inactive periglandular and/or stromal endometrial fibrosis including glandular alterations within fibrotic foci. Single glands and/or glandular nests can be affected [3]. It is not clear if hormonal changes, during reproductive cycle, are involved in the progress of this disease [4]–[7].

In order to provide more precise diagnosis and to characterize phenotypic variations of the uterus surface of mares with endometrosis, the expression pattern of selected endometrial proteins such as steroid hormone receptors, protein of proliferation intensity (Ki-67-antigen), the filaments vimentin, desmin, α-smooth muscle actin (α-actin), laminin and others have been studied [7]–[9]. These studies demonstrated that affected equine endometrium seems unable to provide an appropriate environment for the correct expression of these proteins when compared with healthy endometrium [7], [8]. However, until now the etiology of endometrosis is not defined and no effective treatment is available.

MSCs can be isolated from different adult sources and bone marrow and adipose tissue are more commonly used in researches. These cells have the capacity to differentiate into several tissues of mesoderm and ectoderm origin including bone, cartilage, tendon, muscle, adipose and neurons. MSCs secrete a diverse set of bioactive molecules which are immunomodulatory [10], [11]. Other molecules released by MSCs provide regeneration and remodeling of injured tissue through their trophic activities [12], [13], which involve inhibition of apoptosis, stimulation of MSC-mediated angiogenesis by secretion of VEGF, as well as anti-scar formation [14]. Finally, MSCs secrete mitogens which stimulate tissue-intrinsic progenitors to divide and appropriately differentiate [15], [16]. Thus, proposed clinical application of MSCs suggested their use as medicinal signaling cells which can be used as site-regulated, multidrug dispensaries, or “drugstores” able to promote and support the natural regeneration of focal injuries [13].

Previously, we isolated and successfully expanded *in vitro* multipotent equine adipose tissue–derived mesenchymal stem cells (eAT-MSCs) which present significant proliferative rate [17]. These cells were able to differentiate efficiently into mesodermal derivatives such as bone, cartilage and adipose tissue. Undifferentiated state and differentiation capacity of eAT-MSCs are maintained even after cryopreservation [17]. Based on current knowledge about the expression pattern of selected endometrial proteins, we aimed at analyzing the capacity of allogeneic eAT-MSCs previously isolated by our group to influence the expression of these proteins in mares’ endometrial tissue leading to positive local remodeling. It is important to highlight that in present work we are not tending to evaluate clinical effect of these cells once clinical studies will be our next challenge.

## Materials and Methods

### Animals

All studies were approved by the ethical committee of the School of Veterinary Medicine and Animal Science, University of Sao Paulo, SP, Brazil (Protocol 1804/2009). Six cyclic and healthy mares of various breeds, aging between 6 and 21 years with different degrees of endometrosis were used. These mares were part of an experimental herd and were maintained at the Faculty of Veterinary Medicine, Federal University of Rio Grande do Sul, in an open field supplemented with oats and alfalfa hay with ad libitum access to water.

An endometrial biopsy of each mare was taken immediately prior to the infusion of our cells and used to classify the degree of endometrosis. Three animals were classified as grade IIb and other three as grade III [18]. A mare from each grade was used as control. Mares were examined for reproductive soundness including evaluation of perineal conformation, transrectal palpation and ultrasound of genital tract, vaginal examination with speculum, bacteriological cultures and endometrial cytology. Only clinically normal mares with negative cytology and negative cultures were used.

Time of estrus was synchronized with prostaglandin F2α (Lutalyse 5 mg im–Pharmacia Brasil Ltda., Sao Paulo, SP, Brasil) and it was confirmed by the presence of a dominant follicle (≥35 mm), uterine edema (2–3) and no corpus luteum at the time of cells transplantation.

### Cells

Equine adipose tissue-derived multipotent mesenchymal stem cells (eAT-MSC) previously isolated and characterized by our group [17] were used. These cells have been cryopreserved in liquid nitrogen for two years. Cells were thawed and used immediately for fluorescent labeling and transplantation. Before cells transplantation and after labeling, eAT-MSC were tested by trypan blue assay and the viability (∼98%) of them was confirmed.

### Fluorescent eAT-MSCs Labeling

To label the cells, Vybrant CFDA SE Cell Tracer Kit fluorescent-nanocristal dye (green) (Invitrogen, Carlsbad, CA, USA; V12883) was used. CFDA SE 10 mM stock solution was prepared immediately prior to use by dissolving the contents of one vial (Component A) in 90 µL of high-quality DMSO provided in the kit (Component B). Next, stock solution was diluted in phosphate-buffered saline (PBS) untilthe desired working concentration of 25 µM was reached. eAT-MSCs were thawed just before staining and washed twice in DMEM-HG. Cell pellets were obtained by centrifugation (100×g, 5 min) and the supernatant was aspirated. Next, eAT-MSCs were gently resuspended in pre-warmed (37°C) PBS containing the probe and then incubated for 15 minutes at 37°C. Cells were re-pelleted by centrifugation and resuspended in 20 ml of fresh pre-warmed 0.9% physiologic solution for further uterine infusion into the mares.

### Experimental Cell Transplantation

The procedure of eAT-MSCs infusion was performed during synchronized estrus according as previously reported [19]. After cleaning the perineal area, the operator wearing a sterile insemination glove, introduced a disposable insemination pipette through the cervix to the uterus body. In order to avoid eventual contaminants, the gloved hand was placed over the tip of the pipette during its introduction into the vagina. At this time, the pipette was guided toward the tip of the right horn helped by rectal palpation. The pipette was connected to the syringe containing 2×10^7^ cells diluted in 20 ml of 0.9% sodium chloride through a sterile rubber connector. The plunger of the syringe was slowly depressed introducing 10 ml of cells suspension. Then, the free end was placed in the left uterine horn and the remainder (10 ml of cells suspension) was infused. Immediately, a second syringe containing 3 ml of 0.9% sodium chloride was coupled to sterile pipette infused in order to ensure the total injection of the volume contained in the pipette and in the connector. The pipette was slowly withdrawn from the vagina.

The two controls mares were infused with 20 ml of 0.9% sodium chloride (10 ml in each horn) performing the same technique used to cell infusion.

Biopsies from the uterine body and both horns from treated and control mares were collected 7, 21 and 60 days after infusion. A total of 60 endometrial uterine biopsies from the six mares, were analyzed by four different pathologists in a blind manner. Uterine biopsies were fixed in 10% buffered formalin, embedded in paraplast, sectioned at 4–5 µm and stained with Hematoxylin and Eosin (HE). Degree of endometrosis was analyzed according to [3] and [2], [20], [21]. All specimens showed signs of endometrosis varying in quantity and degree (mild to severe).

### Immunohistochemistry

The peroxidase anti-peroxidase (PAP) method was used for immunohistochemistry. Tissue sections were mounted on superfrost slides (Life Science Int. GmbH, Frankfurt/Main, Germany). Paraffin wax sections were rehydrated and endogenous peroxidase activity was inhibited by 3% H2O2 (30 min). Primary antibodies were diluted in TBS (Tris-buffered saline) with 1% BSA (bovine serum albumin). Depending on the antibody, different dilutions and pretreatments were applied and are summarized in Table 1. All of these primary antibodies used in our research have been previously used in mares [8]. Primary monoclonal antibody crossreacting with mouse antihuman CK18 as well as polyclonal antibodies rabbit antihuman Ki-67-antigen, rabbit antimouse laminin, rabbit antihuman fibronectin and goat antihuman vimentin were incubated at 4°C overnight. Negative control sections were treated with only secondary antibodies diluted in BSA. Rat antimouse (Dianova GmbH, Hamburg, Germany) and pig antirabbit IgG (Dako Diagnostika GmbH, Hamburg, Germany) were used as secondary antibodies and, as PAP-complex, served 1∶500 diluted mouse PAP (Dianova GmbH, Hamburg, Germany) and rabbit PAP (Dako Diagnostika GmbH, Hamburg, Germany), respectively. Both were incubated at room temperature for 30 min. Slides were developed in DAB (diaminobenzidinetetrahydrochloride - Fluka Feinchemikalien Neu Ulm, Germany) and counterstained with HE. In order to interpret immunohistochemical results of the fibrotic foci, healthy endometrial structures within the same specimens were used as controls. Protein expression was detected using a Carl Zeiss Axioplan fluoromicroscope (LSM 410, Zeiss, Jena, Germany). Digital images were acquired with CCD camera (Applied Imaging model ER 339) and documentation system used was Cytovision v. 2.8 (Applied Imaging Corp. - Santa Clara, CA, USA).

**Table 1:** Antibodies used in immunohistochemistry.

*Primary antibodies*	*Host*	*Type*	*Dilution*	*Source*
α-Actinin^1^	Mouse	Monoclonal	1∶200	Chemicon, CA, USA
CD10^1^ (clone 56C6)	Mouse	Monoclonal	1∶25	AbCam, San Francisco, USA
Cytokeratin 18^1^	Mouse	Monoclonal	1∶200	Cell Marque, CA, USA
ER^1^ (clone 14C8)	Mouse	Monoclonal	1∶100	Thermo Scientific, CA, USA
Ki-67^1^	Rabbit	Polyclonal	1∶100	Santa Cruz Biotechnology, CA, USA
Laminin^1^	Rabbit	Polyclonal	1∶25	AbCam, San Francisco, USA
Vimentin^2^ (clone C20)	Goat	Polyclonal	1∶50	Santa Cruz Biotechnology, CA, USA

1 IgG polyclonal goat anti-mouse + goat anti-rabbit HRP (secondary antibody).

2 Polyclonal rabbit anti-goat HRP (secondary antibody).

### Confocal Microscopy

Images were collected using an LSM 510 (Zeiss) laser scanning confocal microscope and all slides were analyzed by four different pathologists in a blind manner. FITC was excited by argon-ion laser set at 488 nm and the emitted light filtered using a 505-nm (FITC) long pass filter. Sections were taken at approximately the mid-height level of the cells.

### Ki-67 Expression Analysis

Ki-67 positive cells density may not be uniform in the biopsy; therefore epithelial cell proliferation rate in the glands was assessed by Ki-67 stained cells, as previously described by [22], [23] in 250 cells of five different fields by two different specialists in a blind manner. The mean called proliferation index (P) and pattern deviation (Mean ± SD) as well as positive stained nuclear cells in the 5 fields were calculated. Differences between treated and untreated animals are shown in percentage.

## Results

### Transplantation and Homing of eAT-MSCs

**Figure 1:**
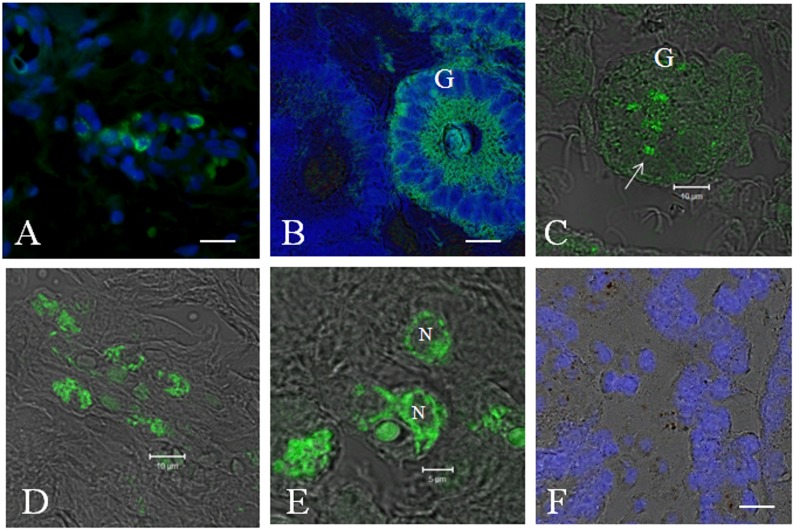
Homing of eAT-MSCs (green fluorescence) after transplantation in endometrium of mares with endometrosis. A) Homing of eAT-MSCs in periglandular space B) eAT-MSC contribution into the whole uterine gland (G). White arrow indicates uterine gland without eAT-MSCs. C) Incorporation of several eAT-MSC (white arrow) into uterine gland epithelia. D–E) eAT-MSCs localization in periglandular space. N-Nucleus. F) Control animals injected with only saline solution. A, B, F = nucleus stained by DAPI (blue). Confocal microscopy: Fluorescence (Fcm) + Digital Interference Contrast (DIC). A–B = Fcm. C–F = Fcm + DIC. Scale bars: A, C, D = 10 µm; B, E, F = 5 µm.

For eAT-MSCs transplantation in utero, cells (∼2×10^7^) were placed in a catheter and infused immediately. In order to confirm allogeneic eAT-MSCs homing in endometrial tissue, direct fluorescence was used. The presence of fluorescently labeled eAT-MSCs (green) in mares’ uterus was observed seven days after intrauterine cells infusion (Figure 1A–E). Figure 1F represents control mare infused with saline solution. eAT-MSCs were visualized in periglandular space (Figure 1A, D, E) as well as in single glands (Figure 1B, C) in three mares. One of the mares which presented advanced degree of chronic degenerative endometrosis, did not show any eAT-MSCs engraftment (data not shown).

### Immunohistochemical Expression Study of Proteins before and after eAT-MSC Transplantation

**Figure 2:**
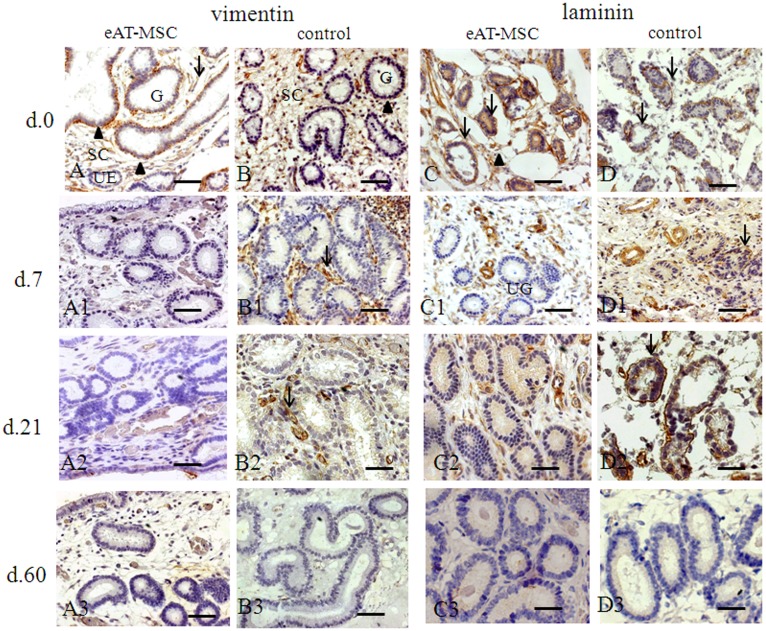
Vimentin and laminin expression before (at day 0) and after (at days 7, 21 and 60) eAT-MSCs intrauterine transplantation: A–A3 and D–D3 - experimental; B–B3 and D–D3 - control. A, B) At day 0, vimentin (black arrowheads) localized in damaged epithelia of glands (G) and in fibrotic stromal cells (SC, black arrows). Unaffected epithelia (UE) showed no signs of vimentin expression. A1–A3) At days 7, 21 and 60, the absence of vimentin expression. B1, B2) Vimentin expression is still observed (black arrows) at days 7 and 21, in control. B3) At day 60, control mares showed no signs of vimentin expression. C, D) At day 0, laminin demonstrated high discontinuity of epithelial basal lamina (black arrows) and a diffuse intracytoplasmatic laminin expression in metabolic active fibrotic stromal cells (black arrowheads); C1, C2) At days 7 and 21, unaffected glands (UG) with a diffuse intracytoplasmatic laminin expression were observed. C3) At day 60, the absence of vimentin expression was shown. D1, D2) At days 7 and 21, control maintains same pattern of laminin expression as in (D). D3) At day 60, laminin expression was not visualized in control. Light Microscopy (LM). Scale bars: A–C2 = 50 µm; C3, D2, D3 = 25 µm.

**Figure 3:**
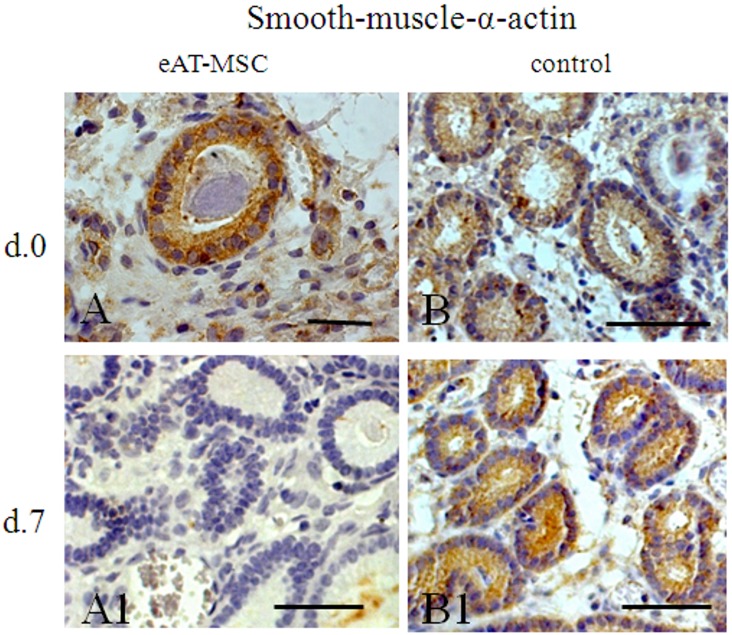
Smooth-muscle-α-actin (SMA) expression before (at day 0) and after (at day 7) eAT-MSCs intrauterine transplantation. A, B) At day 0, SMA expression was observed in uterine glands (white arrowhead) and in periglandular fibroblasts (black arrow). A1) At day 7, SMA showed no signs of expression. B) Pattern of SMA expression is similar to A and B. LM. Scale bars: A = 25 µm; A1, B, B1 = 50 µm.

**Figure 4:**
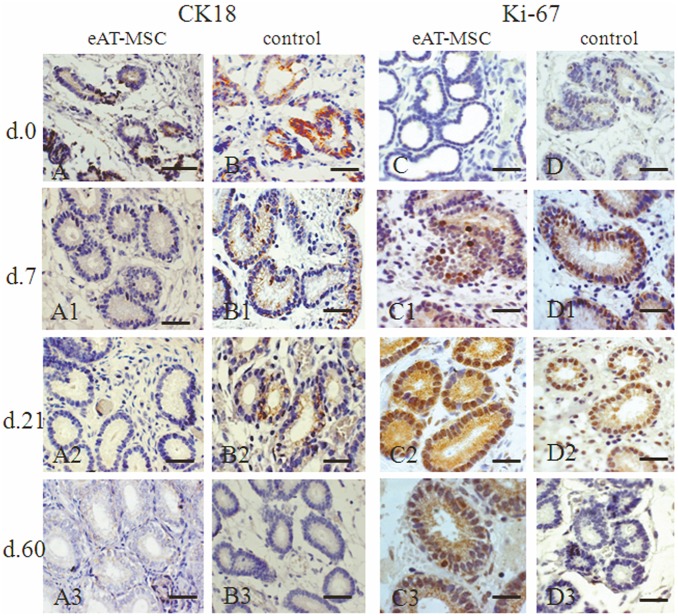
Cytokeratin 18 (CK18) and Ki-67 expression before (at day 0) and after (at days 7, 21 and 60) eAT-MSCs intrauterine transplantation: A–A3 and D–D3 - experimental group; B–B3 and D–D3 - control. A, B) At day 0, CK18 (black arrows) localized in damaged epithelia of glands (G). A1–A3) At days 7, 21 and 60, the absence of CK18 expression was observed. B1, B2) At days 7 and 21, CK18 expression was still observed (black arrows) in control. B3) At day 60, control mares showed no signs of CK18 expression. C, D) At day 0, none or a few Ki-67 positive cells were observed. C1, D1) At day 7, amount of Ki-67 positive cells (black arrow) was increased. C2, D2) At day 21, both groups showed positive Ki-67 staining. C3) At day 60, the expression of Ki-67 was still observed. D3) In control, the absence of Ki-67 expression. LM. Scale bars: A–D1 = 50 µm; C2, C3, D2, D3 = 25 µm.

In order to evaluate the benefits of eAT-MSCs transplantation and the engraftment of these cells in mares’ uterus with endometrosis, the expression pattern of secretory proteins such as vimentin, laminin, Ki-67, smooth-muscle-α-actin and cytokeratin 18 (CK18) has been analyzed (Figures 2–4).

### Vimentin and Laminin

Basal expression of vimentin in fibrotic gland epithelia and in fibrotic stromal cells was observed in experimental (Figure 2A) and in control (Figure 2B) groups before cells transplantation. Such basal expression of vimentin was no longer observed in the glandular epithelia and stromal cells at day 7 after cells transplantation (Figure 2A1) as well as at days 21 and 60 (Figure 2A2, A3). In control group, the expression of vimentin was strong in fibrotic stromal cells at day 21 (Figure 2B1, B2) with no expression at day 60 (Figure 2B3).

Laminin expression was intense and before cell transplantation, a high discontinuity of epithelial basal lamina was noted, as well as, diffuse cytoplasmatic expression in fibrotic stromal cells in all mares (Figure 2C, D). In biopsies obtained from mares which received the cells, diffuse intracytoplasmatic expression of laminin in fibrotic stromal cells, but not in epithelial basal lamina (Figure 2C1, C2) was observed at days 7 and 21, with no expression at day 60 (Figure 2C3). In control group, an atypical expression pattern of laminin (Figure 2D) was maintained until days 7 and 21 (Figure 2D1, D2) and was no longer detected at day 60 (Figure 2D3).

### Smooth-muscle-α-actin

In all mares with endometrosis, the cystic dilated glands were coated by a distinct layer of cells positive for anti-α-SMA (Figure 3A, B). Additionally, this protein expression was detected in fibroblasts surrounding fibrotic uterine glands (Figure 3A). At day 7, in biopsies from mares which received eAT-MSCs, the expression of anti-α-SMA in glands, was no longer evidenced (Figure 3A1), while in control animals the expression of α-SMA was still present (Figure 3B1). At days 21 and 60, the expression of this protein was no longer detected in both groups (data not shown).

### Cytokeratin 18

According to previous studies, cytokeratins are known to be expressed in epithelial glands of normal equine endometrium [24]. However, co-expression of cytokeratin and vimentin in mares’ endometrium is exclusively associated with fibrotic areas or pathologically inactive endometrium [25]. Hence, CK18 expression was also evaluated. Similar to vimentin (Figure 2A, B), CK18 was expressed in uterine glands of all animals before cells transplantation (Figure 4A, B). Starting from day 7, this protein expression was no longer observed in experimental group (Figure 4A1–A3), while in control group it was still expressed in uterine glands at day 7 and 21 (Figure 4B1–B2), but not at day 60 (Figure 4B3).

### Ki-67-antigen

In Figure 4(C–D3) the expression of Ki-67 antigen in uterine glands and in periglandular stromal cells is presented in both, experimental (Figure 4C–C3) and control groups (Figure 4D–D3). At day 0, none or few Ki-67 positive cells were observed in both groups (Figure 4C, D). At day 7, both groups showed an increased quantity, but still a small amount of Ki-67 positive cells (Figure 4C1 and D1). At day 21, Ki-67 positive cells significantly increased in both experimental (Figure 4C2), and control (Figure 4D2) groups. At day 60, two groups (experimental and control) had registered progressive decrease of proliferative cells in glands (Figure 4C3, D3). These qualitative data was confirmed by quantitative analysis of Ki67 positive cells in glands (Table 2).

**Table 2:** Proliferation rate analyzed by Ki-67 antigen expression.

*Groups*	*0 Days*	*7 Days*	*21 Days*	*60 Days*
**Experimental Group n = 3**	4.09%+/−1.08%	10.46%+/−3.95%	67.04%+/−4.8%	25.95%+/−5.09%
**Control Group n = 2**	9.46%+/−5.01%	17.08%+/−4.45%	21.03%+/−6.71%	3.29%+/−2.09%

### Histological Characterization of Early Positive Remodeling of Endometrium

**Figure 5:**
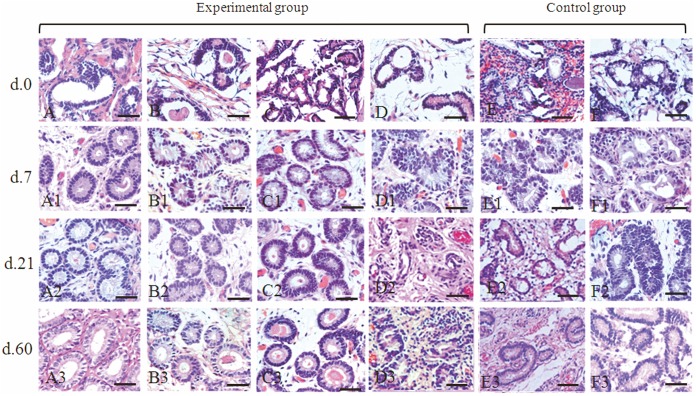
Histological analysis of alterations in mares’ endometrium following eAT-MSCs intrauterine transplantation. A–D3) Mares which received the cells. DE–F3) Control mares. A–F) Morphology of endometrial surface prior eAT-MSCs intrauterine cells transplantation. A1–D1) Day 7 after eAT-MSCs intrauterine transplantation. A2–D2) Same as in (A1–D1) at day 21. A3–D3) Same as in (A1–D1) at day 60. E1–F3) Respective controls. LM. Scale bars: A–F3 = 50 µm.

Morphological characteristics of biopsies obtained from mares with endometrosis are presented in Figure 5 A–F. In accordance with [8] the foci of endometrosis, including periglandular stromal cells (“fibrotic stromal cells”) and affected glandular epithelia, as well as, neighbored unaltered glands, were taken into consideration. In all animals, single glands and/or glandular nests were affected. Following eAT-MSCs transplantation, positive histological changes were observed in three mares (Figure 5 A1–C3). One mare, which presented more severe degree of endometrosis and received the cells, did not show cells engraftment into endometrium (data not shown) as well as morphological improvement (Figure 5D–D3). Control animals show a relatively unaltered pattern of endometrial histology (Figure 5E1–F3).

## Discussion

Endometrosis in mares is characterized by periglandular or stromal fibrosis associated with epithelial alterations in fibrotic glands [1], [2]. Such epithelial and stromal mal-differentiation in mares’ endometrial causes an altered protein expression pattern which results in a disturbed microenvironment, causing poor embryonic nutrition and early embryo death [26]. Contradictory results have been published concerning the expression of steroid hormone receptors during endometrosis and it is not clear if the expression of selected proteins is direct related with hormonal changes [8]. MSCs are known to promote tissue re-modeling and regeneration through multiple mechanisms such as engraftment, differentiation, immunomodulation and diverse trophic activities [13]. In order to evaluate the effects of MSCs on an altered endometrial microenvironment in mares during breeding season, allogeneic eAT-MSCs were transplanted into the uterus of mares with endometrosis. Herein, we provided for the first time, evidence that atypical morphological and functional differentiation of glandular and periglandular endometrial stromal cells have changed after eAT-MSCs transplantation into the uterus. According to [8], such altered protein expression pattern of vimentin, laminin, smooth-muscle-α-actin and CK18 was observed in endometrium of mares, thus confirming endometrosis.

The incorporation of these cells following intrauterine transplantation was demonstrated using direct immunofluorescence. MSCs migrated to the periglandular space and in few cases contributed significantly to glandular epithelia improvement. As shown previously, the co-expression of cytokeratin and vimentin is normal during the proliferation phase in human but not in equine endometrium [27]–[29]. The co-expression of both of these proteins in epithelial cells has been observed in different tumors and for many years CK18 has been recognized as an epithelial marker in histopathological diagnostic [30], [31]. Accordingly, we observed the co-expression of these proteins in all mares’ endometrium with endometrosis. Following eAT-MSCs transplantation, the co-expression of these markers was no longer observed in mares’ endometrium when compared with control group, suggesting positive effect of eAT-MSCs transplantation on the expression pattern of these proteins.

The analysis of expression patterns of proteins as α-SMA, laminin and Ki-67 antigen support aforementioned data. It has been shown that stromal cells of destructive endometrosis, in particular in the active destructive endometrosis, tended to express more α-SMA [7]–[8]. It is of common knowledge that local stimuli induce smooth muscle differentiation in resident fibroblasts and neighboring epithelial or mesenchymal cells can produce these stimuli [32]. Accordingly, differentiation of periglandular cells to myofibroblasts, leading to a comparable histopathology, was also reported for fibrotic dilated glands of the human endometrium [33]. In our study, the expression of microfilament α-SMA in uterine glands was observed before eAT-MSCs transplantation in all studied animals, which is in accordance with previous studies [7]–[8] and well known paracrine effects of MSC [13]. However, at day 7, the expression of α-SMA was no longer observed in uterine glands of animals which received eAT-MSCs.

Laminin is known to be the major protein in the basal lamina which is a protein network foundation for most cells and organs. Laminin influences cellular differentiation, migration, adhesion as well as phenotype and survival [34]. After eAT-MSCs transplantation, atypical laminin localization, which observed in mares with endometrosis [8], was positively modified in experimental group at day 7 while in control endometrium such alteration did not occur. Atypical laminin localization in both groups before eAT-MSCs transplantation can be explained by the fact that myofibroblasts are known to build up an incomplete layer of basal lamina on their cell surface which is known to maintain smooth muscle cells in a differentiated state [30].

Ki-67 antigen is an excellent marker to determine the growth cell fraction of a given cell population. An altered intensity of cell proliferation (which also depends on the steroid cycle) within the fibrotic foci during the estrous cycle was shown to be obvious [8]. The effect of extrinsic human MSCs on the viability, proliferation and differentiation of intrinsic cells in the local of injury was demonstrated over past years [12]–[13]. Accordingly, our data demonstrated that the amount of Ki-67 positive cells was significantly higher in endometrium of mares treated with eAT-MSCs in comparison with untreated animals (Table 2).

At day 7 after eAT-MSCs transplantation, early morphological remodeling of endometrium was observed when compared with untreated mares. Morphological alterations in endometrium were escorted by changes in the expression pattern of analyzed proteins in treated animals but not in control mares which continue to show atypical protein expression pattern until day 21. Taken together, our data provides evidence of morphological and functional benefits of MSCs transplantation into mares with endometrosis.

The number of empty seasons is one of the factors that can influence the incidence of endometrosis. The fact that one animal with advanced degree of endometrial degeneration has not responded to the treatment could suggest the preventive use of stem cells therapy which can slow down the degeneration process that occurs with mares which failed to be pregnant in previous breeding season.

## Conclusion

It is noteworthy that allogeneic eAT-MSCs which were cryopreserved during two years in liquid nitrogen can be used directly after thawing without additional culturing *in vitro*. These cells can be transplanted without application of immunosuppressive protocols while presenting successful and efficient homing in mares’ endometrium. Additionally, following intrauterine transplantation, eAT-MSCs were able to induce early (at day 7) and prolonged (until day 60) positive remodeling of endometrial tissue of these mares with endometrosis. These extrinsic allogeneic eAT-MSCs were able to stimulate local environment composed by epithelial and periglandular stromal cells and to modulate the expression of cytokeratin, vimentin, α-SMA and laminin, thus avoiding further development of pathological processes which leads to the formation of fibrotic regions on horse endometrium.

Our data suggest that these cells similar to human MSCs from bone marrow, act through multiple mechanisms such as homing in fibrotic periglandular and glandular space, modulation of the expression pattern of studied proteins and increase glandular epithelial cells proliferation thus providing anti-scar effect.

It is important to note, that local therapy is designed to prevent a local recurrence of the injury. Our study targets a local effect of MSCs on injury which takes place in endometrium of mares. Therefore, logic rationality suggests that the combination between local and systemic stem cell therapies may provide more efficient tool to combat endometrosis, one of the major cause for equine infertility.
